# HIV prevalence and risk factors in infants born to HIV positive mothers, measured by dried blood spot real-time PCR assay in Tigray, Northern Ethiopia

**DOI:** 10.1186/s12887-019-1636-y

**Published:** 2019-07-26

**Authors:** Mulu Lemlem Desta, Muthupandian Saravanan, Haftamu Hilekiros, Atsebaha Gebrekidan Kahsay, Nesredin Futwi Mohamed, Alefech Addisu Gezahegn, Bruno S. Lopes

**Affiliations:** 1 Department of Medical Microbiology and Immunology, Division of Biomedical Science, School of Medicine, College of Health Science, Mekelle University, 1871, Mekelle, Ethiopia; 2 Tigray Health Research Institute (THRI), 1871, Mekelle, Ethiopia; 3 Department of Medical Microbiology, School of Medicine, Medical Sciences and Nutrition, University of Aberdeen, 0:025 Polwarth Building, Aberdeen, AB25 2ZD UK

**Keywords:** Prevalence of HIV, Risk factor, HIV exposed infants, DBS, RT-PCR

## Abstract

**Background:**

Infants infected during pregnancy or while breastfeeding requires early HIV diagnosis at 6 weeks after birth to identify HIV infection and timely treatment. The objective of this work was to determine the prevalence and associated risk factors of HIV among HIV exposed infants in the Tigray regional state, Northern Ethiopia.

**Methods:**

A cross-sectional study was conducted on 350 exposed infants born to HIV seropositive mothers from September 01 to December 30, 2016. Convenient consecutive sampling technique was employed to enroll HIV exposed infants from age 6 weeks to 18 months attending prevention of mother to child transmission (PMCT) clinic at Anti Retroviral Therapy (ART) site facility in Tigray, Ethiopia. Sociodemographic data and associated risk factors were collected using a structured questionnaire. Dried Blood Spot (DBS) samples were collected from each infant and transported by post to Tigray Health Research Institute to detect HIV infection using real-time Polymerase Chain Reaction (PCR). Data were entered into EPI Info version 7, exported and analyzed using Statistical Package for Social Sciences (SPSS) version 22. *p*-value less than 0.05 was deemed to be statistically significant by Fisher’s exact test.

**Results:**

Three hundred forty infants (175 males, 165 females) met the criteria for selection during the completion of the study and the overall HIV prevalence was found to be 2.1% (*n* = 7). The majority of infants were from urban areas (*n* = 246, 72.4%). 45.5% (5/11, *p* = 0.001) infants were without ARV prophylaxis, 60% (3/5, *p* = 0.001) infants born to mothers who did not take maternal PMTCT intervention, 43% (3/7, *p* = 0.001) infants born to mothers who were not enrolled to ART care, and 6.1% (4/66, *p* = 0.029) infants of unmarried mothers showed statistically significant difference.

**Conclusions:**

The overall prevalence of HIV among exposed infants was high but lower than the Millennium Development Goal targets. In order to eliminate the mother to child HIV transmission (MTCT) ARV prophylaxis in infants must be strengthened, and enrollment of HIV positive pregnant women to PMTCT and ART care and treatment is needed.

## Background

HIV induced acquired immunodeficiency syndrome (AIDS) pandemic, has been a major medical and public health problem globally [1]. According to the World Health Organization (WHO), an estimated number of 39 million people have died since the first cases were reported in 1981 [2]. There were approximately 36.7 (34.0–39.8) million people living with HIV with 2.1 (1.8–2.4) million people becoming newly infected in 2015 globally (http://www.unaids.org/sites/default/files/media_asset/UNAIDS_FactSheet_en.pdf). Sub-Saharan Africa is the most affected region, with 25.6 (23.1–28.5) million people living with HIV in 2015 and accounts for almost 70% of the global prevalence (http://www.unaids.org/sites/default/files/media_asset/UNAIDS_FactSheet_en.pdf, http://www.who.int/mediacentre/factsheets/fs360/en/). The contribution of children under 15 infected by HIV was 2.6 million with 88% of the cases being reported from Sub-Saharan Africa (http://www.unaids.org/sites/default/files/media_asset/UNAIDS_FactSheet_en.pdf, https://data.unicef.org/wp-content/uploads/2015/12/2015-Children-Adolescents-and-AIDS-Statistical-Update-Executive-Summary_244.pdf).

HIV/AIDS epidemic remains one of the important public health challenges in Ethiopia with the first cases of HIV and AIDS recognized in 1984 and 1986 respectively [3]. According to the ministry of health report, the overall estimated national HIV prevalence was 1.14%, where 769,600 people were living with HIV and about 15,700 with new HIV infections, while 35,600 AIDS-related deaths were recorded at the end of 2014 (http://www.afro.who.int/countries/ethiopia). The prevalence of HIV related estimate for Tigray, Ethiopia was predicted at 1.8% for 2017 with 6055 cases in the 0–14 age group and 58,742 cases in 15 and above age group (https://www.ephi.gov.et/images/pictures/download2009/HIV_estimation_and_projection_for_Ethiopia_2017.pdf).

The majority of HIV infection among children under the age of 15 years is due to MTCT which can occur during pregnancy (in the uterus), labor and delivery, and breastfeeding (http://www.unaids.org/sites/default/files/media_asset/UNAIDS_FactSheet_en.pdf, [4]). In the absence of any intervention, the risk of MTCT during pregnancy, delivery, and breastfeeding is estimated to be 25 to 45% [5, 6]. Every day there are nearly 1500 new infections in children less than 15 years of age, more than 90% of them occurring in the developing world (http://www.who.int/hiv/pub/guidelines/paediatric020907.pdf?ua=1).

HIV infected infants are at increased risk of life-threatening infections such as pneumonia due to *Pneumocystis carinii*, *Mycobacterium tuberculosis*, and nutritional deficiencies and other infections, such as malaria and diarrhea, which are often complicated to treat and are the leading cause of infant mortality in HIV-infected newborns and infants [6].

Following the WHO recommendations, in Ethiopia, the rapid scale-up of ART provides access for pregnant women living with HIV and has averted more than 900,000 new HIV infections among children since 2009 [5]. A recent report from Tigray Regional Health Bureau showed that the prevalence of HIV in exposed infants has declined consistently from a peak of 9% in 2010/11 to 3.1% in 2014/15 [7]. HIV transmission rate from mother to child at 6 weeks decreased from 19% in 2009 to 10% in 2013 with the final HIV transmission rate from mother to child, including during breastfeeding decreasing from 39 to 25% during the same year period. On the contrary, the number of women (15–49 years old) acquiring HIV increased by 74% since 2009 which increased from 4500 in 2009 to 7800 in 2013 (http://www.unaids.org/sites/default/files/media/documents/UNAIDS_GlobalplanCountryfactsheet_ethiopia_en.pdf). Since 2001, due to the launch of a national program for PMTCT of HIV infection by the Ethiopian Ministry of Health, the number of facilities with PMTCT service has reached 1,445 providing ARV prophylaxis for 10,302 HIV positive pregnant women and 4,945 exposed infants in 2011 [8]. The prophylactic medication coverage during pregnancy has improved significantly in east and southern Africa but it is still lower than the 80% target because of limited healthcare provision during childbirth [8].

To further reduce and prevent the risk of MTCT and have an AIDS-free generation, early virological testing is critical for identifying infected infants and to strengthen the quality of HIV-exposed infant follow-up (http://www.ilo.org/wcmsp5/groups/public/%2D%2D-ed_protect/%2D%2D-protrav/%2D%2D-ilo_aids/documents/legaldocument/wcms_125389.pdf). Positive HIV antibody test in an infant indicates maternal but not necessarily infant HIV infection [9].

National guidelines in Ethiopia recommends that infants exposed to HIV should be tested by polymerase chain reaction (PCR) for the detection of viral nucleic acid at 6 weeks of age using dried blood spot samples wherever the facilities and resources for these assays are available (http://www.ilo.org/wcmsp5/groups/public/%2D%2D-ed_protect/%2D%2D-protrav/%2D%2D-ilo_aids/documents/legaldocument/wcms_125389.pdf). Almost all regional states in Ethiopia are currently implementing this programme. However, a scientific study that can clearly show the prevalence and associated risk factors for exposed infants in Tigray region has not been previously performed. Hence, the aim of this study was to assess the prevalence and associated risk factors of HIV among exposed infants in Tigray, Northern Ethiopia.

## Methods

### Study area and sample size determination

The study was conducted in 83 public ART site health facilities in Tigray Regional State, Northern Ethiopia spread across 54,572.6 sq. km. As projected from the 2007 census, the region has an estimated total population of 5,151,998 [10]. The region has 240 public health facilities serving the population of Tigray and neighboring areas of other regions. The region has one specialized hospital, 15 general hospitals and 224 health centers; of these 117 are governmental ART site health institutions. The sample size (*n* = 350) was determined using a single population proportion formula. A non-probability convenient consecutive sampling technique was employed to enroll the study participants. The number of study participants in each zone were allocated proportionally to its size using the proportional allocation formula.

### Study participants and study variables

A cross-sectional study was employed among all HIV exposed infants from the age of 6 weeks and less than 18 months, attending PMTCT clinic in the governmental ART site health facilities from September 01 to December 30, 2016. Exposed infants who were critically ill and infants whose parents were unwilling to give their consent were excluded from the study.

### Data collection

Clinical data such as CD4 count of the mother during delivery, infant birth weight and treatment history (eg: infant ARV prophylaxis at birth, maternal PMTCT intervention) was collected from infant medical record and mother’s history recording charts using a checklist. Socio-demographic data such as age, sex, residence, occupation and other risk factors of the study participants were collected for each study participants from mothers using a structured questionnaire by trained personnel.

#### Laboratory analysis

##### Sample collection, handling, and transportation

Capillary blood sample (~50ul) was collected from each HIV-exposed infant via a heel or toe skin prick with sterile lancet by a trained nurse in the PMTCT clinic using pre-punch protein Saver 903® cards (Whatman Ltd., Piscataway, USA), with at least 4 spots in the same location, which was then be left to dry by putting the card horizontally. The dried cards were packed individually with desiccant sachets and humidity indicators in each PMTCT clinic at ART site health facilities until further processing. The specimens were transported to the Tigray health research institute (testing site) by the post and received within two to 3 days and processed immediately upon receipt. If there was an unavoidable delay at the health facility or in the testing site, cards were stored at 15–30 °C for less than 12 weeks and 2–8 °C or − 10 °C for period greater than 12 weeks [11].

##### Sample processing using DNA PCR

**Fig. 1:**
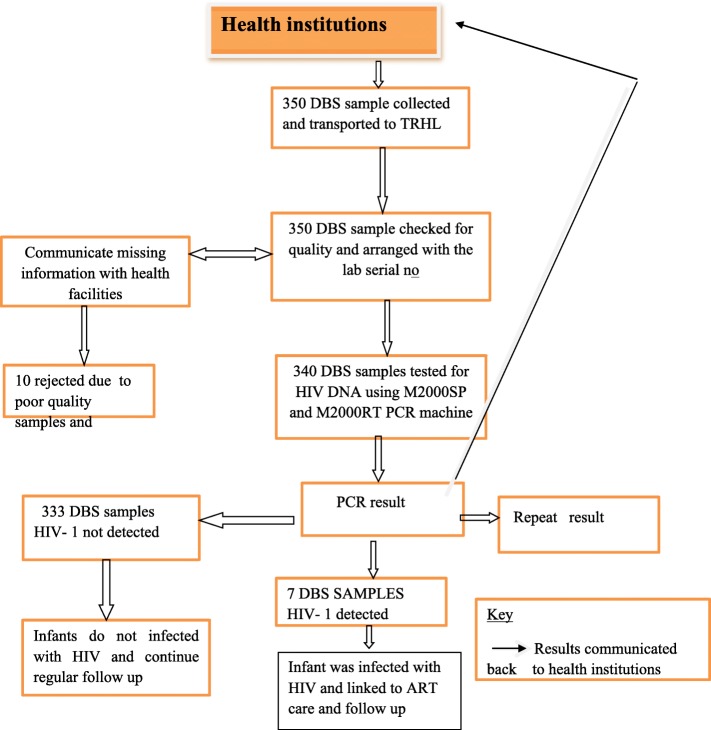
Workflow in detection of infant HIV infection by PCR

Upon the arrival of sample at Tigray health research institute, it was processed using a standard procedure. Two spots of DBS samples (~ 50 μL each) were transferred into a 50 ml falcon tube with 1.7 mL of bulk lysis buffer; then incubated for 20 min at room temperature with intermittent gentle mixing in between 10 min. The whole volume was transferred into a 5 ml reaction vessel: samples and controls were placed in sample racks with one positive control (2G31X) and one negative (2G31Z) controls, and reagents placed on different rack within the bar-coded reaction vessels on Abbot EID M2000 sample preparation (Abbot EID M2000sp, United States of America) automation for extraction process (Fig. 1).

The Abbott m2000sp then performed the auto method nucleic acid extraction, washing, and elution. The internal control (IC) was introduced in the mLysis buffer before the mLysis buffer was loaded on the Abbott M2000sp. After the sample extraction was completed, the amplification reagents (the master mix tube) were loaded on the Abbott m2000sp. The master mix was then added to the 96-well Optical Reaction Plate with the extracted nucleic acids on the Abbott m2000sp and centrifuged at 3900 rpm using centrifuge (Sigma 2–16, Germany) for 5 min to avoid bubble and transferred in to real-time PCR machine (Abbot EID m2000rt, United States of America) for DNA amplification and detection. When amplification was completed, Abbott m2000rt analyzed the Real-time PCR data and assigned a qualitative positive or negative result to each sample. The HIV-1 target sequence that is present at the amplification cycle is detected by the use of fluorescent-labeled oligonucleotide probes by the Abbott m2000rt instrument. The probes do not generate signal unless they are specifically bound to the amplified product.

### Quality assurance

Questionnaire was first prepared in English and translated into Tigrigna (local language). Training was provided to data collectors on sample collection before prior to data collection. The collected data were reviewed for completeness, accuracy, clarity, and consistency by data collectors and the principal investigator. A questionnaire was checked and manually updated if there were incomplete or unrecorded values, and unlikely responses and laboratory results were recorded in the laboratory data result in formats coded for each participant.

Abbott real-time HIV-1 qualitative controls (high positive and negative) were used to establish run validity of the Abbott real-time HIV-1 qualitative assay when used for the qualitative detection of HIV-1 nucleic acid from human dried blood spots (DBS) in each batch.

### Data analysis and interpretation

Each collected data was labeled using specific patient codes. The data were entered into Epi Info version 7. Data was then exported and analyzed using Statistical Package for Social Sciences (SPSS) version 22. Descriptive statistics were computed to summarize data and result was presented using tables. Polymerase chain reaction results were analyzed using frequency and percentage. Association between different variables with outcome was analyzed using Fisher exact test. A *p*-value less than 0.05 was considered as statistically significant.

## Results

### Socio-demographic characteristics of study participants

The response rate of the present study was 97.1% (340/350). About 143 (42%) of study participants were enrolled from hospitals and the remaining 197 (58%) were from the ART health centers. Among the 340 infants that participated participant, 175 (51.5%) were males and the rest females. Majority of HIV exposed infant’s mothers (*n* = 234, 68.8%) were in the age range of 25–34 years. More than half of the mothers (*n* = 211, 62.1%) were educated at least at the primary level or above and 274 (80.6%) of the mothers were married and 246 (72.4%) belonging to an urban area (Table 1).

**Table 1:** Exposed infant and maternal-related characteristics by HIV positivity among HIV exposed infants in Tigray, Ethiopia, 2016 (*n* = 340)

Variables	Frequency, [n, %]	HIV status		*P*-value*
		Positive, [n, %]	Negative, [n, %]	
Gender of infant				
Male	175 (51.5)	3 (1.7)	172 (98.3)	
Female	165 (48.5)	4 (2.4)	161 (97.6)	0.71
Residence				
Urban	246 (72.4)	6 (2.4)	240 (97.6)	
Rural	94 (27.6)	1 (1.1)	93(98.9)	0.68
Maternal education				
Illiterate	129 (37.9)	5 (3.9)	124(96.1)	
Primary & above	211 (62.1)	2 (0.9)	209 (99.1)	0.11
Maternal age (in years)				
18–24	56 (1.5)	1 (1.8)	55 (98.2)	
25–34	234 (68.8)	5 (2.1)	229 (97.9)	
35–44	50 (14.7)	1 (2.0)	49 (98)	NA
Maternal marital status				
Not married	66 (19.4)	4 (6.1)	62 (93.9)	
Married	274 (80.6)	3 (1.1)	271 (98.9)	0.029*
Maternal occupation				
Housewife	260 (76.5)	1(0.4)	259 (99.6)	
Government employee	17 (5)	1 (5.9)	16 (94.1)	NA
Self-employed	63 (18.5)	5 (7.9)	58 (92.1)	
Infant age at enrolment				
6 weeks-6 months	338 (99.4)	7 (2.1)	331 (97.9)	
6–18 months	2 (0.6)	0 (0)	2 (100)	NA
Place of delivery				
Institutional	332 (97.6)	7 (2.1)	325 (97.9)	
Home	8 (2.4)	0 (0)	8 (100)	NA
Infant Feeding pattern				
Exclusive breast-feeding	337 (99.1)	7 (2.1)	330 (97.9)	
Formula	2 (0.6)	0 (0)	2 (100)	
Mixed feeding	1 (0.3)	0 (0)	1 (100)	NA
Mode of delivery				
Normal	323 (94.4)	7 (2.2)	316 (97.8)	
Cesarean	17 (5.6)	0 (0)	17 (100)	NA
Infant birth weight (Kg)				
< 2.5	15 (4.4)	0 (0)	15 (100)	
≥ 2.5	325 (95.6)	7 (2.2)	318 (97.8)	NA
Infant ARV prophylaxis				
Not Received	11 (3.2)	5 (45.5)	6 (54.5)	
Received	329 (96.8)	2 (0.6)	327 (99.4)	0.001*
Maternal ART enrolment				
Enrolled	333 (97.9)	4 (1.2)	329 (98.8)	
Not enrolled	7 (2.1)	3 (42.9)	4 (57.1)	0.001*
PMTCT				
Option B+ and on ART	335 (69.1)	4 (1.2)	331 (98.8)	
None	5 (1.5)	3 (60)	2 (40)	0.001*
Maternal CD4^+^ count at late pregnancy (cell/mm^3)^				
< 200	13 (3.9)	0 (0)	13 (100)	
≥ 200	327 (96.1)	7 (2.1)	320 (97.9)	NA

*P-Value < 0.05 indicates significant result by Fisher's exact test

The majority, 338 (99.4%) of the exposed infants were in the age range of 6 weeks to 6 months and were born (*n* = 332, 97.6%) in various health institutions in Tigray. The mean age of infants at sample collection time was 49.9 days (SD + 27.8). Almost all, 337 (99.1%) of the infants were exclusively breastfed, 323 (94.4%) of them had a normal delivery, 325 (95.6%) had a birth weight of greater than or equal to 2.5 kg and 329 (96.8%) received ARV prophylaxis (Table 1). In addition, higher proportions 235 (69.1%) of the mothers were taking ‘Option B+’ (Table 1).

### Prevalence of infant HIV using RT PCR

Three hundred and forty HIV-exposed infants were tested for HIV infection by real-time PCR. This study revealed that the overall prevalence of HIV infection among exposed infants was 2.1% (*n* = 7) (Table 1). Moreover, all the 7 HIV-infected infants were from institutional births, exclusively breastfeed and were normal deliveries in the age group of thier mothers 25–34 years.

### Risk factors associated with HIV positivity in HIV exposed infants

In this study, maternal marital status, ART care enrollment, taking PMTCT intervention and receiving infant ARV prophylaxis were showed statistically significant with HIV positivity (Table 1). Higher proportions of HIV positive infants were from non-married mothers 4 (6.1%) and not being married was a risk factor deemed to be statistically significant (*P* = 0.029) for infants contracting HIV. In addition, the proportion of HIV positivity was higher in infants whose mothers were not enrolled to ART care than their enrolled counterparts (6.1% vs. 1.1%) and was statistically significant (*P* = 0.001) risk factor. The infants who did not receive ARV prophylaxis had statistically higher proportions of HIV positivity 45.5%, (*p* = 0.001). In this study HIV positivity was higher proportion in infants from urban area than the rural area but was not a statistically significant risk factor (*p*-value = 0.678).

## Discussion

The prevalence of HIV infection in this study was 2.1% (7/340) and comparable to that observed in Brazil (2.01%) [12]. Our findings were within the global and national plan to reduce MTCT rates to 5% or less by 2015 [2], (http://pdf.usaid.gov/pdf_docs/PA00JWM5.pdf). The findings of the current study were higher than studies conducted in Ukraine (1.6%) [13] and France (1.5%) [14]. The difference may be due to high coverage of PMTCT interventions in developed countries and limited access, lack of awareness, poor quality of service and others in resource-limited countries like Ethiopia. The prevalence in the current study was however; lower than the study reports from Brazil (11.8%) [15], Kenya (5%) [16], Malawi (4.1%) [17] and Zambia (6.5%) [18]. The difference in prevalence might be explained due to the difference to ART and PMCT follow up, awareness to HIV, policies, and strategies on HIV control and prevention, methodology and sample size.

Prevalence in this study was lower than those conducted in other parts of Ethiopia including Southern Ethiopia (4.16%) [19], Bishoftu hospital (4.3%) [20], North West Ethiopia (10%) [21], Jimma (10.9%) [22], Driedawa (15.7%) [8] and Addis Ababa (32.1%) [23]. These studies showed higher prevalence, as most of these are retrospective studies their data collection time were ranging from 2005 to 2013, before the implementation of successful intervention strategies like Option B+. Since 2013, Ethiopia is implementing suitable guidelines such as Option B+ where a lifelong antiretroviral treatment is provided to all pregnant and breastfeeding women living with HIV regardless of CD4+ count or WHO clinical stage and Nevirapine to infants in the first 6 weeks of life [24]. In the current time, HIV infection in exposed infants is decreasing through time due to the overall efforts are done to prevent new HIV infection in infants.

Vertical transmission of HIV in infants is a multi-factorial process, which involves factors associated with HIV-1 transmission. In the present study, the prevalence of HIV was high among infants who did not take antiretroviral prophylaxis at birth (*n* = 11, 3.2%), than those who did take ARV prophylaxis at birth (*n* = 329, 96.8%) and this was found to be statistically significant (*p* < 0.05). Our findings are in line with studies reported from Driedawa [8], Malawi [17], Zambia [18], Nigeria [25] and Ukraine [13]. This result is consistent with the widely scientifically accepted fact that ARV in infants decreases the risk of HIV infection [26].

In addition to infant’s prophylaxis taking maternal PMTCT intervention was statistically significant in decreasing HIV positivity. The finding was agreed with the study done in North West Ethiopia [21], Amhara [27], Tanzania [28], Malawi [17], Nigeria [25] and Zambia [18]. Accordingly, infants who were born to mother who took Option B+ and already on ART had a lower rate of HIV positivity (1.2%) than those who born to mother who didn’t take PMTCT intervention that was (60%). This is supported by the scientifically accepted idea that maternal PMTCT interventions decrease the HIV positivity in exposed infants [29]. The lower prevalence of HIV (1.2%) in pregnant women was also reported previously in the Amhara region in Ethiopia, which reported high prevalence (10.1%) among the infants born to these mothers [27]. This was due to delayed HIV diagnosis, inadequate use of antiretroviral therapy and lack of skilled delivery, which promotes mother-to-child transmission of HIV. In the current study the HIV status of HIV positive mothers enrolled for ART was very low (1.2%, 4/333) (Table 1). This is due to the improved coverage at public health facilities responsible for PMTCT and also low number of sample size.

The HIV positivity was high (10.1%) among HIV exposed infants of mothers with the age range of 25–34 which is consistent with a study from Amhara, Ethiopia [27]. In addition, HIV positivity was high in mothers with CD4^+^count >200cell/mm^3^compared to mothers with CD4^+^count of <200cell/mm^3^ in our study. In Zimbabwe [30] it was observed that maternal CD4^+^cell count less than 200 cell/mm^3^ was significantly associated with infant HIV positivity. But there are other contradictory to reports from France [14], Brazil [15] and Malawi [17]which show no significant association. In the current study as we observe maternal CD4^+^ count of >200cell/mm^3^ in mothers that are HIV positive, we can conclude that infection can be transmitted from these mothers to the exposed infants. This may be supported with a conclusion from a study with effective antiretroviral coverage; maternal CD4 count does not affect the HIV positivity [31]. Our result showed that due to many national and international efforts towards PMTCT and HIV infection, the HIV prevalence among exposed infants is decreasing from time to time.

## Conclusion

The overall prevalence of HIV among exposed infants was 2.1%, which is still high prevalence but lower than the Millennium Development Goal (MDG) targets. We observed that, HIV positivity was higher in infants who did not take ARV prophylaxis and whose mothers did not enroll to ART care and follow up and infants of mothers who did not take PMTCT interventions during pregnancy or childbirth. Therefore, in order to eliminate the MTCT, increasing antenatal HIV screening and linking HIV positive pregnant women to PMTCT and ART care and providing appropriate ARV prophylaxis to infants must be done efficiently. Strengthening national monitoring surveillance, coverage, and quality of HIV interventions in mother and child health (MCH) services is important for the PMTCT program.

The prevalence of HIV among infants born from seropositive mothers is reduced to the meaningful number (< 5%) because of the appropriate measures taken for reducing the transmission of HIV from mothers to infants. But, because a significant number of infants from seropositive mothers are still infected. We recommend: Strengthening of the PMTCT of HIV programme, increasing antenatal HIV screening and linking it to care and treatment of HIV positive mothers to obtain zero infant HIV prevalence in the region. Infant prophylaxis and maternal PMTCT interventions should be provided to all exposed infants and mothers based on the guidelines by the health institutions. ART centers in every health institutes and Tigray regional health bureau HIV regional offices should work together to enroll all HIV seropositive mothers to ART care and support which will help to decrease the prevalence of HIV in Tigray even further.

### Limitation of the study

We were unable to see the independent effect of variables on the outcome so further studies taking an adequate period of time and on larger sample size is required.

## Data Availability

The datasets used and/or analyzed during the current study are available from the First author and corresponding author on reasonable request.

## Abbreviations

AIDS: Acquired immunodeficiency syndrome
ANC: Antenatal care
ART: Antiretroviral Therapy
ARV: Anti-retroviral
CD4: Cluster of Differentiation- 4
CDC: Centers for Disease Control and Prevention
EBF: Exclusive breastfeeding
HAART: Highly active antiretroviral therapy
HIV: Human Immunodeficiency Virus
MCH: Mother and Child Health
MF: Mixed feeding
MTCT: Mother-to-child transmission;
NAT: Nucleic amplification test
PCR: Polymerase chain reaction
PMTCT: Prevention of Mother-to-child transmission
WHO: World Health Organization

## References

[1] Lal RB, Chakrabarti S, Yang C (2005). Impact of genetic diversity of HIV-1 on diagnosis, antiretroviral therapy & vaccine development. Indian J Med Res.

[2] UNAIDS (2011). Global plan towards the elimination of new HIV infections among children by 2015.

[3] Hladik W, Shabbir I, Jelaludin A, Woldu A, Tsehaynesh M, Tadesse W (2006). HIV/AIDS in Ethiopia: where is the epidemic heading?. Sex Transm Infect.

[4] Lu D, Liu J, Samson L, Bitnun A, Seigel S, Brophy J, Leonard L, Remis RS (2014). Factors responsible for mother-to-child HIV transmission in Ontario, Canada, 1996-2008. Can J Public Health.

[5] Chi BH, Adler MR, Bolu O, Mbori-Ngacha D, Ekouevi DK, Gieselman A, Chipato T, Luo C, Phelps BR, McClure C, Mofenson LM, Stringer JS (2012). Progress, challenges, and new opportunities for the prevention of mother-to-child transmission of HIV under the US President's emergency plan for AIDS relief. J Acquir Immune Defic Syndr.

[6] Gangar J (2009). Nutritional Assessment of Newborns of HIV Infected Mothers. Indian Pediatr.

[7] Barnabas G, Pegurri E, Selassie HH, Naamara W, Zemariam S (2014). The HIV epidemic and prevention response in Tigrai, Ethiopia: a synthesis at sub-national level. BMC Public Health.

[8] Wudineh F, Damtew B (2016). Mother-to-child transmission of HIV infection and its determinants among exposed infants on care and follow-up in Dire Dawa City**,** East Ethiopia. AIDS Res Treat.

[9] Siberry GK (2014). Preventing and managing HIV infection in infants, children, and adolescents in the United States. Pediatr Rev.

[10] World Health Organization (2014). Global update on the health sector response to HIV, 2014.

[11] Mei Joanne (2014). Dried Blood Spot Sample Collection, Storage, and Transportation. Dried Blood Spots.

[12] Alcântara KC, Lins JBA, Albuquerque M, Aires LM, Cardoso LPV, Minuzzi AL, Stefani MMA (2012). HIV-1 mother-to-child transmission and drug resistance among Brazilian pregnant women with high access to diagnosis and prophylactic measures. J Clin Virol.

[13] Bailey H, Semenenko I, Pilipenko T, Malyuta R, Thorne C (2010). Ukraine European collaborative study group: factors associated with abandonment of infants born to HIV-positive women: results from a Ukrainian birth cohort. AIDS Care.

[14] Tubiana R, Le Chenadec J, Rouzioux C, Mandelbrot L, Hamrene K, Dollfus C, Faye A, Delaugerre C, Blanche S, Warszawski J (2010). Factors associated with mother-to-child transmission of HIV-1 despite a maternal viral load< 500 copies/ml at delivery: a case-control study nested in the French perinatal cohort (EPF-ANRS CO1). Clin Infect Dis.

[15] Martínez A, VPd H, ALd S, Mendoza-Sassi R, Von Groll A, Soares EA, D'avila N, Silveira J, Leal RG, Tanuri A (2006). Determinants of HIV-1 mother-to-child transmission in Southern Brazil. An Acad Bras Cienc.

[16] Coovadia HM, Rollins NC, Bland RM, Little K, Coutsoudis A, Bennish ML, Newell M (2007). Mother-to-child transmission of HIV-1 infection during exclusive breastfeeding in the first 6 months of life: an intervention cohort study. Lancet.

[17] Kim MH, Ahmed S, Preidis GA, Abrams EJ, Hosseinipour MC, Giordano TP, Chiao EY, Paul ME, Bhalakia A, Nanthuru D (2013). Low rates of mother-to-child HIV transmission in a routine programmatic setting in Lilongwe, Malawi. PLoS One.

[18] Torpey K, Kasonde P, Kabaso M, Weaver MA, Bryan G, Mukonka V, Bweupe M, Zimba C, Mwale F, Colebunders R (2010). Reducing pediatric HIV infection: estimating mother-to-child transmission rates in a program setting in Zambia. J Acquir Immune Defic Syndr.

[19] Tadele T, Tamiso A, Tadele T (2014). Incidences and predictors of HIV positivity among infants who born from HIV positive mother who have follow up at two hospitals of southern Ethiopia, 2014. Sci J Public Health.

[20] Olana T, Bacha T, Worku W, Tadesse BT (2016). Early infant diagnosis of HIV infection using DNA-PCR at a referral center: an 8 years retrospective analysis. AIDS Res Ther.

[21] Koye DN, Zeleke BM (2013). Mother-to-child transmission of HIV and its predictors among HIV-exposed infants at a PMTCT clinic in Northwest Ethiopia. BMC Public Health.

[22] Wondafrash B, Hiko D (2016). Dried blood spot test for HIV exposed infants and children and their anti-retro viral treatment status in selected hospitals in Ethiopia. Ethiop J Health Sci.

[23] Amare H, Weldesenbet Z, Tsadik AG, Ayalew E, Aragaa R, Hassen F, Desta K, Tsegaye A (2014). Prevalence and risk factors of HIV infection among infants, born from HIV seropositive mothers, tested by DNA-PCR at yekatit 12 Hospital, Addis Ababa, Ethiopia. Int J Pharm Sci Res.

[24] World Health Organization (2015). Guideline on when to start antiretroviral therapy and on pre-exposure prophylaxis for HIV. Guideline on when to start antiretroviral therapy and on pre-exposure prophylaxis for HIV.

[25] Anoje C, Aiyenigba B, Suzuki C, Badru T, Akpoigbe K, Odo M, Odafe S, Adedokun O, Torpey K, Chabikuli ON (2012). Reducing mother-to-child transmission of HIV: findings from an early infant diagnosis program in south-south region of Nigeria. BMC Public Health.

[26] US Public Health Service Task Force (2013). Recommendations for use of antiretroviral drugs in pregnant HIV-1-infected women for maternal health and interventions to reduce perinatal HIV-1 transmission in the United States.

[27] Berhan Z, Abebe F, Gedefaw M, Tesfa M (2014). Prevalence of HIV and associated factors among infants born to HIV positive women in Amhara Region, Ethiopia. Int J Clin Med.

[28] Kilewo C, Karlsson K, Ngarina M, Massawe A, Lyamuya E, Swai A, Lipyoga R, Mhalu F, Biberfeld G (2009). Mitra Plus study team: prevention of mother-to-child transmission of HIV-1 through breastfeeding by treating mothers with triple antiretroviral therapy in Dar Es Salaam, Tanzania: the Mitra Plus study. J Acquir Immune Defic Syndr.

[29] McIntyre J (2005). Prevention of mother-to-child transmission of HIV: treatment options. Expert Rev Anti-Infect Ther.

[30] Ngwende S, Gombe NT, Midzi S, Tshimanga M, Shambira G, Chadambuka A (2013). Factors associated with HIV infection among children born to mothers on the prevention of mother to child transmission programme at Chitungwiza Hospital, Zimbabwe, 2008. BMC Public Health.

[31] Nyamagoudar A, Mruthyunjaya S, Patil M, Banapurmath C (2017). Study on impact of maternal CD4 count on birth outcomes and mother to child transmission of HIV infection. Int J Community Med Public Health.

